# An innovative targeted therapy for fluoroscopy-induced chronic radiation dermatitis

**DOI:** 10.1007/s00109-021-02146-3

**Published:** 2021-10-23

**Authors:** Kai-Che Wei, Shih-Fan Lai, Wei-Lun Huang, Kuo-Chung Yang, Ping-Chin Lai, Wan-Ju Wei, Tsung-Hsien Chang, Yun-Chen Huang, Ya-Chuan Tsai, Shin-Chih Lin, Sun-Jang Lin, Shih-Chieh Lin

**Affiliations:** 1 Department of Dermatology, Kaohsiung Veterans General Hospital, Kaohsiung, Taiwan; 2 Department of Cosmetic Applications and Management, Yuhing Junior College of Health Care and Management, Kaohsiung, Taiwan; 3 Department of Dermatology, National Yang Ming Chiao Tung University, Taipei, Taiwan; 4 Department of Biomedical Engineering, College of Medicine and College of Engineering, National Taiwan University, Taipei, Taiwan; 5 Division of Radiation Oncology, Department of Oncology, National Taiwan University Hospital and College of Medicine, Taipei, Taiwan; 6 Department of Radiation Oncology, Kaohsiung Veterans General Hospital, Kaohsiung, Taiwan; 7 Department of Plastic and Reconstructive Surgery, Kaohsiung Veterans General Hospital, Kaohsiung, Taiwan; 8 The Kidney Institute and Division of Nephrology, China Medical University Hospital, Taichung, Taiwan; 9 Department and Graduate Institute of Microbiology and Immunology, National Defense Medical Center, Taipei, Taiwan; 10 Institute of Basic Medical Sciences, College of Medicine, National Cheng Kung University, Tainan, Taiwan; 11 Department of Physiology, College of Medicine, National Cheng Kung University, Tainan, Taiwan; 12 Department of Dermatology, National Taiwan University Hospital and College of Medicine, Taipei, Taiwan; 13 Research Center for Developmental Biology and Regenerative Medicine, National Taiwan University, Taipei, Taiwan; 14 Institute of Molecular Medicine, College of Medicine, National Cheng Kung University, Tainan, Taiwan

**Keywords:** YAP1, Radiation dermatitis, Glucocorticoid receptor, Prednisolone, Exosome; Fluoroscopy-guided intervention

## Abstract

**Abstract:**

Fluoroscopy-induced chronic radiation dermatitis (FICRD) is a complication of fluoroscopy-guided intervention. Unlike acute radiation dermatitis, FICRD is different as delayed onset and usually appears without preexisting acute dermatitis. Unfortunately, the chronic and progressive pathology of FICRD makes it difficult to treat, and some patients need to receive wide excision and reconstruction surgery. Due to lack of standard treatment, investigating underlying mechanism is needed in order to develop an effective therapy. Herein, the Hippo pathway is specifically identified using an RNA-seq analysis in mild damaged skin specimens of patients with FICRD. Furthermore, specific increase of the Yes-associated protein (YAP1), an effector of the Hippo pathway, in skin region with mild damage plays a protective role for keratinocytes via positively regulating the numerous downstream genes involved in different biological processes. Interestingly, irradiated-keratinocytes inhibit activation of fibroblasts under TGF-β1 treatment via remote control by an exosome containing YAP1. More importantly, targeting one of YAP1 downstream genes, nuclear receptor subfamily 3 group C member 1 (NR3C1), which encodes glucocorticoid receptor, has revealed its therapeutic potential to treat FICRD by inhibiting fibroblasts activation in vitro and preventing formation of radiation ulcers in a mouse model and in patients with FICRD. Taken together, this translational research demonstrates the critical role of YAP1 in FICRD and identification of a feasible, effective therapy for patients with FICRD.

**Key messages:**

• YAP1 overexpression in skin specimens of radiation dermatitis from FICRD patient.

• Radiation-induced YAP1 expression plays protective roles by promoting DNA damage repair and inhibiting fibrosis via remote control of exosomal YAP1.

• YAP1 positively regulates NR3C1 which encodes glucocorticoid receptor expression.

• Targeting glucocorticoid receptor by prednisolone has therapeutic potential for FICRD patient.

**Supplementary Information:**

The online version contains supplementary material available at 10.1007/s00109-021-02146-3.

## Introduction

Radiation dermatitis is the most common type of radiation damage. The severity of radiation injury increases with accumulated exposure [1]. In addition to radiotherapy, radiation exposure accompanied with fluoroscopy-guided interventional therapeutic procedures such as percutaneous coronary intervention (PCI) can cause radiation damage [2]. When performing PCI, cardiology interventionalists can visualize coronary arteries without performing open surgery by utilizing radiation. More than 1 million cases of fluoroscopy-guided interventions are done annually in the USA [3], and the number of complex interventional procedures has increased steadily. However, there are only few studies reporting the epidemiology of radiation skin damage following fluoroscopy-guided interventions, and the incidence remains unclear.

Fluoroscopy-guided interventional procedures can cause acute radiation dermatitis and fluoroscopy-induced chronic radiation dermatitis (FICRD). FICRD is different from acute radiation dermatitis in manifestation and clinical course. Acute radiation dermatitis occurs within hours or days up to 90 days after radiation exposure [4, 5]. Topical or systemic corticosteroids are supposedly effective by suppressing inflammation. In contrast, the onset of FICRD is delayed and occurs more than 90 days even years after radiation exposure. Most cases with FICRD have no history of transient erythema or preexisting acute radiation dermatitis. Patients usually present with progressive worsening of skin lesion with severe pain or pruritus. Although there are no skin surface defects at the first clinical presentation, it typically manifests later as extensive fibrosis of the entire dermis due to unknown mechanisms [6], and minor injury in FICRD could subsequently lead to non-healing ulceration. This type of ulcer is difficult to treat and many of them require wide excision and reconstruction surgery to heal [6, 7]. Ideally, a safe drug is warranted to treat or prevent FICRD from deterioration in a long-term treatment course [4]. Nowadays, there is no recommended effective medical therapy.

Evidence has shown that ionizing radiation causes an increase in reactive oxygen species-(ROS)-induced oxidative stress and DNA damage to human cells [8]. Cells have to remove these hazardous situations by activation of several well-known cellular protection mechanisms, including antioxidants, radical scavengers, DNA damage sensing, ATM/ATR, and DNA repair systems [9–12] to maintain genome integrity and survive after X-ray exploration. However, the factors integrating these different cellular protection mechanisms have been poorly studied. YAP1, a mediator of the Hippo pathway, has been revealed to be a DNA damage responder in *Saccharomyces cerevisiae* [13]. In addition, YAP1 activation prevents cell apoptosis of urothelial cell carcinomas after irradiation-induced DNA damage [14], and YAP1 inhibition radio-sensitizes triple negative breast cancer cells by disrupting the DNA damage response and cell survival pathways [15]. Furthermore, skin has been known to constantly renew and repair itself throughout adult life through the functioning of epidermal stem cells [16], and YAP1 is a critical modulator of epidermal stem cell proliferation and tissue expansion [17]. These findings suggest that YAP1 plays a potential role in protecting irradiated skin cells. Herein, we demonstrate that YAP1 has a protective role in FICRD.

## Materials and methods

### Patients and clinical specimens

The current study complies with the guidelines of the Declaration of Helsinki and was approved by the Institutional Review Board of Kaohsiung Veterans General Hospital (IRB number: VGHKS17-CT10-07). Detail information was described in the Supplementary Materials.

### RNA-seq and bioinformatics analyses

NGS analyses were performed by using total RNA from the skin specimens, for which the detail procedures are described in the Supplementary Materials. Gene lists from different regions of radiation-induced dermatitis were further analyzed using a GO analysis provided from the DAVID (the Database for Annotation, Visualization, and Integrated Discovery), a bioinformatics tool. Potential downstream target YAP1 genes were identified based on a previous study [18] and their loci containing YAP1-binding sites were analyzed from ChIP-Atlas, an integrative public database providing ChIP-seq data.

### Animal irradiation model

The animal-use protocol was approved by the Institutional Animal Care and Use Committee of National Taiwan University. All mice were housed in the animal facility at National Taiwan University (Taipei, Taiwan). C57BL/6 mice (12-week-old) were purchased from the Taiwan National Laboratory Animal Center. Detailed procedures for the animal study are provided in the Supplementary Materials.

### Statistical analysis

Data was expressed as mean ± standard deviation of the mean, and the statistical analyses were performed using GraphPad Prism 5.0 (GraphPad Software, Inc. La Jolla, CA, USA). Paired or unpaired *t*-tests were used for the group comparisons. Furthermore, a one-way ANOVA followed by a Dunnett analysis was used for more than two groups. A *p* value less than 0.05 was considered statistically significant.

## Results

### The Hippo pathway was a specific event occurring in the skin region with mild damage

**Fig. 1:**
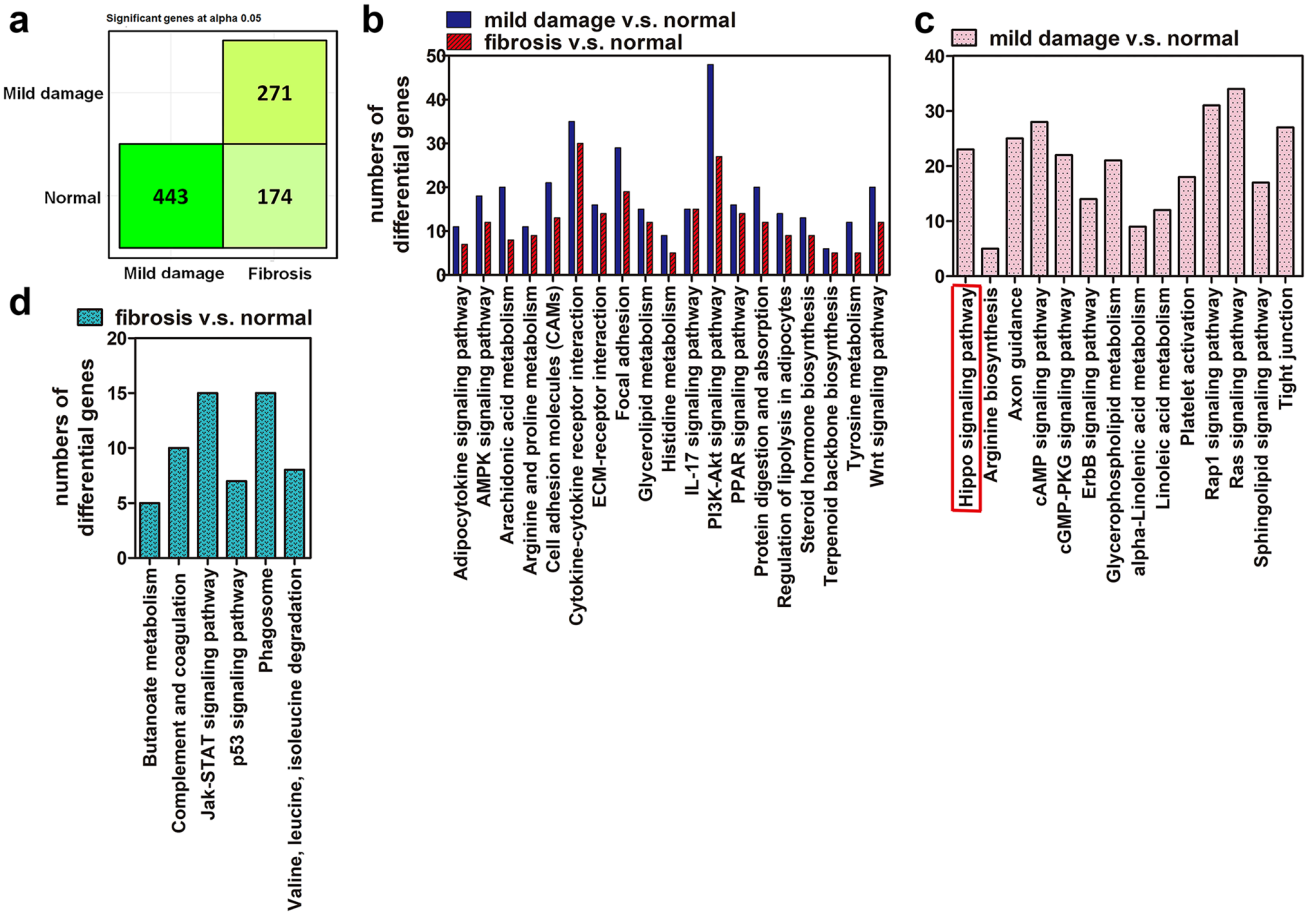
Hippo signaling pathway was a specific event occurring in the skin sample of FICRD with mild damage. (**a**) Tissue samples from normal, mild damage, and fibrosis areas were further analyzed using an RNA-seq technique. A figure was drawn using genes with statistical differences as a summary (*p* < 0.05) of a comparison of normal, mild damage, and fibrosis samples. (**b**–**d**) Genes with statistical differences from mild damaged vs. normal and fibrosis vs. normal were used to performed gene ontology analysis. Common events under both conditions are shown in (**b**), specific events in the mild damage vs. normal damage samples are shown in (**c**), and specific events in the fibrosis vs. normal samples are shown in (**d**)

To identify the potential underlying mechanism causing radiation-induced skin disorder, skin specimens, including normal, mildly damaged, and severely damaged (fibrosis) parts were collected from patients who received their latest PCI 1 year ago. Their skin pathological profiles were validated (Supplementary Fig. 1a–c), and then, RNA-seq analyses were performed. The results showed significant differences in 443 genes in the skin tissue with mild damage (MD) compared to the normal (Nor) tissue, 174 genes in the fibrotic tissue (F) compared to the normal tissue, and 271 genes in the F compared to the MD tissue (Fig. 1a). Next, a gene ontology (GO) analysis was performed on the genes from the MD vs. Nor and on the F vs. Nor tissues to reveal their potential functions. The results showed that common events, including the AMPK, PI3K-AKT, PPAR, IL-17, Wnt signaling pathways, arachidonic acid metabolism, steroid hormone biosynthesis, and cell adhesion molecules occurred under both conditions (Fig. 1b). Furthermore, some specific events occurred in the MD vs. Nor (Fig. 1c) and in the F vs. Nor (Fig. 1d). Since these skin samples were collected after the last irradiation exposure a year prior, specific events in the MD vs. Nor tissue were the focus in order to investigate their roles after irradiation and their subsequent effects on fibrosis. Among them, the Hippo pathway caught our attention due to its critical role in skin physiology [19–22].

### YAP1 was overexpressed in the MD tissue of FICRD

**Fig. 2:**
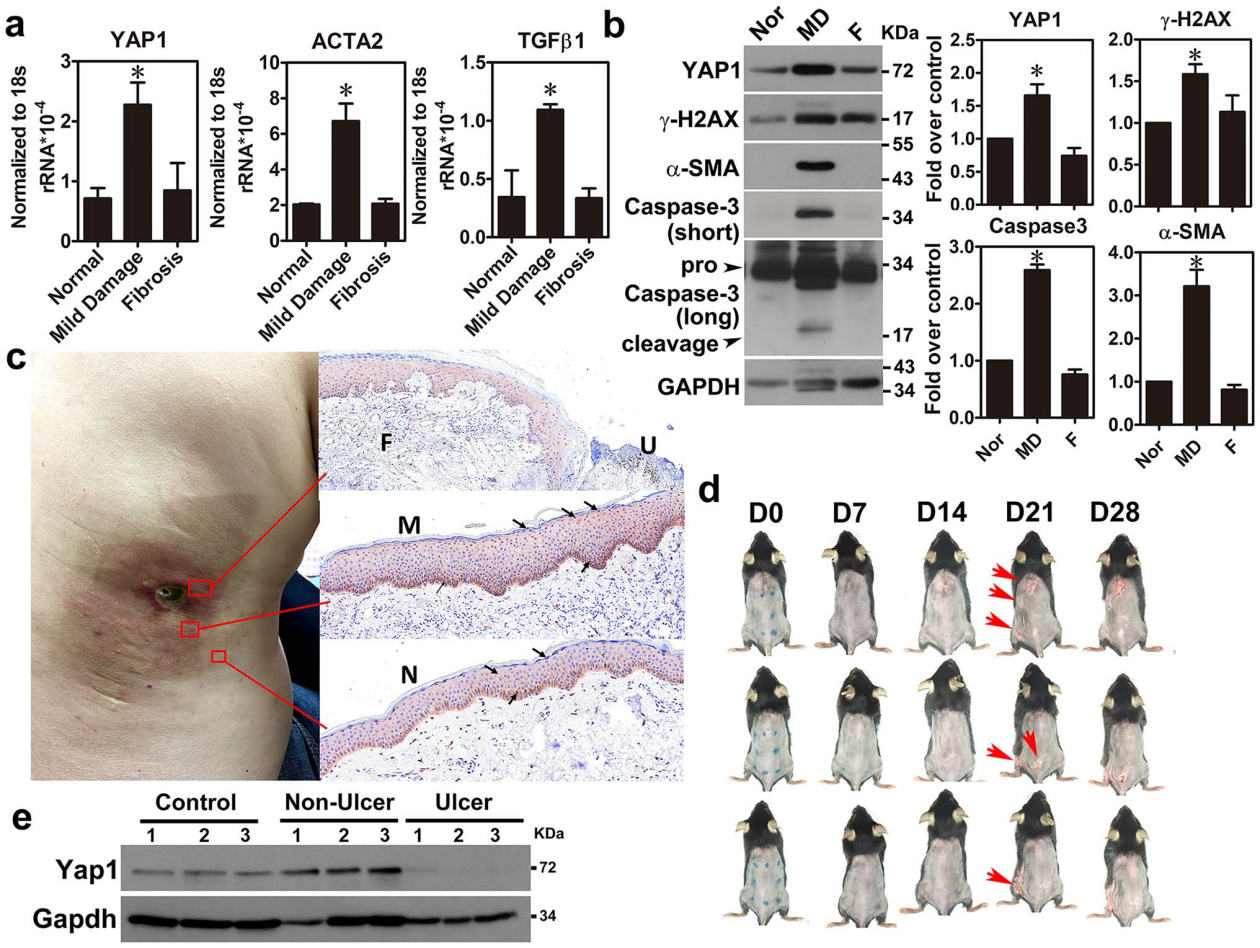
YAP1 was overexpressed in the skin tissue with mild damage. (**a**) YAP1, ACTA2, and TGF-β1 expression levels in the normal, mildly damaged, and fibrotic tissue samples were measured using real-time PCR (*n* = 3). The asterisk indicates *p* < 0.05 obtained using a one-way ANOVA test following Dunnett’s analysis. (**b**) YAP1 expression was measured using a western blot (*n* = 3). Nor, normal tissue; MD, minor damage, F, fibrosis. (**c**) YAP1 protein expression was measured using IHC staining in skin tissues obtained from patients with FICRD (right panel). A representative picture of FICRD related to fluoroscopy-guided intervention. N, normal tissue; MD, mildly damaged tissue; F, fibrosis.; U, ulcer. (**d**) A representative picture of a skin radiation mouse model was shown and incubation time points were annotated as D (Day). Red arrow indicated the wounded region in the mouse skin. (**e**) YAP1 expression was determined by using western blot in skin tissues isolated from a skin radiation of mouse model

To study the role of the Hippo pathway in the MD tissue of patient with FICRD, the YAP1 (a downstream mediator of the Hippo pathway) signature was specifically enriched in the MD tissue after a gene set enrichment analysis (GSEA) (Supplementary Table 1), suggesting that YAP1 may play a role in FICRD. As verification of the bioinformatics findings, the results showed that YAP1 RNA and the protein expression levels were specifically increased in the MD tissues compared to the normal and fibrotic tissues (Fig. 2a, b). In addition, γ-H2AX (a DNA damage marker), the cleavage form of caspase-3 (an apoptosis maker), and α-SMA (a fibrosis marker) were significantly increased in the MD tissue (Fig. 2b), implying that an increase in YAP1 may be related to DNA damage, apoptosis, and fibrosis in vivo. Because skin tissue contained several cell types, YAP1 expression in the normal, MD, and fibrotic skin tissues were also determined using an immunohistochemistry (IHC) staining method. The results revealed that YAP1 expression in the keratinocytes of the epidermis was mainly elevated in the skin region with mild damage and was gradually reduced in the fibrotic region after radiation exposure (Fig. 2c), but not in the other types of cells. Furthermore, similar results were also observed in the mouse model of skin irradiation (Fig. 2d, e). These findings suggest that YAP1 in keratinocytes may play a crucial role in FICRD.

### Irradiation increased YAP1 expression to protect keratinocytes in the skin region with mild damage

**Fig. 3:**
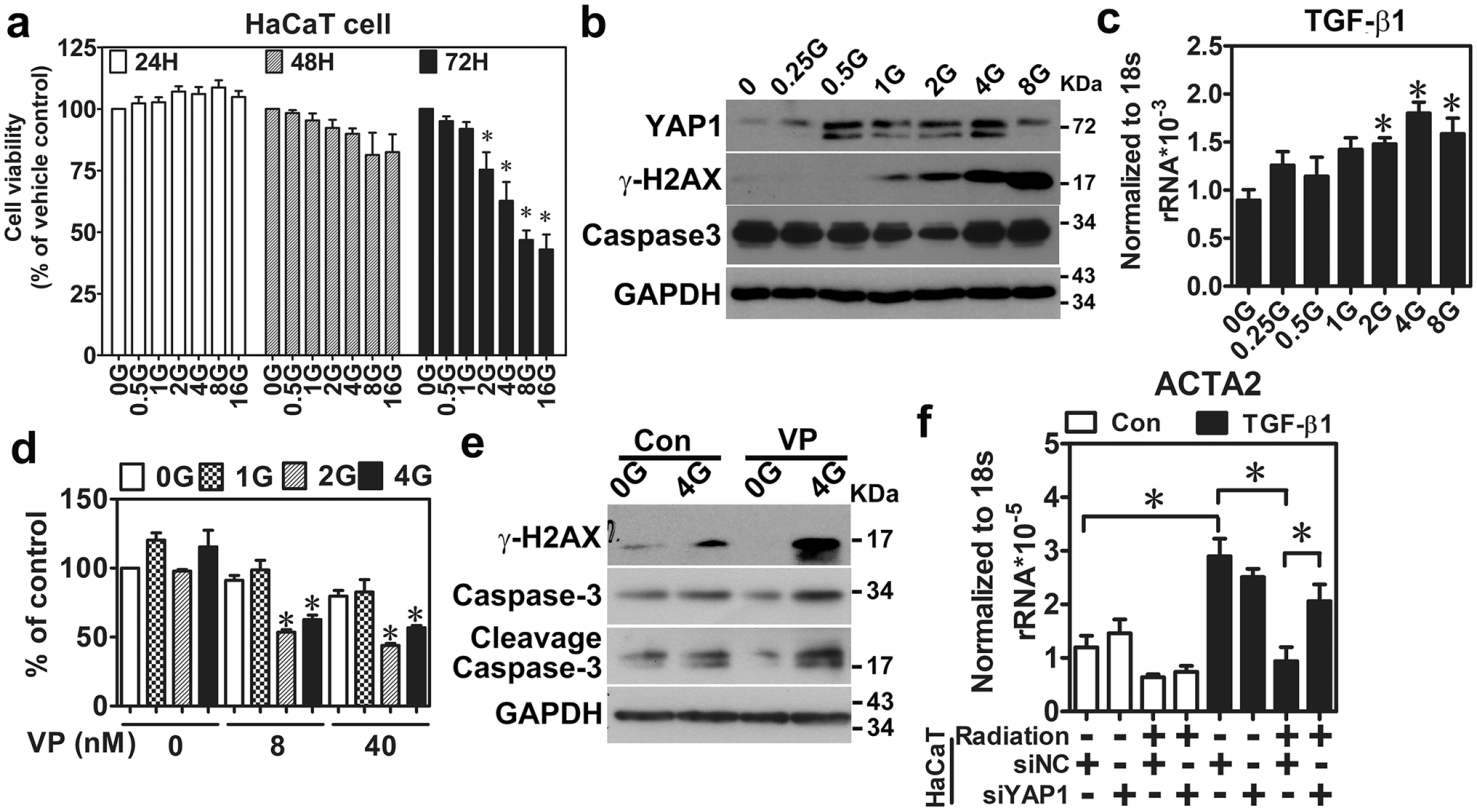
Irradiation-induced YAP1 expression serves a protective role in the pathogenesis of FICRD. (**a**) HaCaT cells having received different doses of radiation and individually incubated for 24, 48, and 72 h were used to measure cell viability. The asterisk indicates *p* < 0.05 using a one-way ANOVA test following a Dunnett’s analysis (*n* = 3). (**b**) YAP1, γ-H2AX, and caspase-3 expression levels were analyzed in HaCaT cells treated with different doses of irradiation after incubation for 24 h. (**c**) TGF-β1 expression level was detected using real–time PCR in HaCaT cells treated with different doses of irradiation after incubation for 24 h (n = 3). (**d**, **e**) HaCaT cells were treated with different doses of a verteporfin (VP) compound for 24 h after irradiation. Cell viability was measured using an MTS assay (*n* = 3) (**d**). An asterisk indicates *p* < 0.05 using a one-way ANOVA test following a Dunnett’s analysis. γ-H2AX and caspase-3 were detected using a western blot (**e**). Con, control; VP, verteporfin. (**f**) HaCaT cells were cocultured with WS1 cells after irradiation and treated with TGF-β1 (5 ng/ml) for 24 h. ACTA2 expression was detected in WS1 cells using real-time PCR (*n* = 3)

To investigate the potential function of YAP1 in FICRD, HaCaT, a human keratinocyte cell line, was treated with different doses of radiation for specific time points. A cell viability assay showed that radiation doses higher than 2 Gy decreased HaCaT cell viability after incubation for 72 h (Fig. 3a). Next, to verify whether irradiation directly contributed to dysregulation of YAP1 levels, YAP1 expression was detected in HaCaT cells after they received different doses of irradiation for 24 h. The results showed that the expression levels of YAP1, γ-H2AX, and TGF-β1 were gradually increased after irradiation with elevated doses (Fig. 3b–c). Furthermore, there was no obvious difference in caspase-3 after irradiation for 24 h, but it was gradually elevated after irradiation for 72 h (Fig. 3b; Supplementary Fig. 2). These results suggest that YAP1 overexpression may be associated with DNA damage, apoptosis, and fibrosis after radiation treatment in vitro. To test this idea, radiation-induced changes in cell viability, DNA damage, and apoptosis markers were measured when YAP1 function was inhibited by its inhibitor, verteporfin (VP), in HaCaT cells. The results showed that VP treatment further reduced cell survival (Fig. 3d), while it increased γ-H2AX and cleavage caspase-3 expression levels in irradiated-HaCaT cells (Fig. 3e). Similar results were observed in YAP1 knockdown by siRNA after irradiation (Supplementary Fig. 3). Next, HaCaT cells with or without the YAP1 knockdown were treated with radiation and then co-cultured with WS1, a fibroblast cell line, where cells receiving or not receiving TGF-β1 treatment were investigated to determine whether YAP1 could potentially be involved in TGF-β1-induced fibroblast activation caused by radiation. Surprisingly, the irradiated-HaCaT cells repressed TGF-β1-induced ACTA2 (α-SMA) expression, while YAP1 knockdown in radiated-HaCaT cells attenuated it (Fig. 3f). Taken together, these results suggest a protective role of YAP1 after irradiation.

### YAP1 positively regulates numerous novel downstream target genes in irradiated keratinocytes

**Fig. 4:**
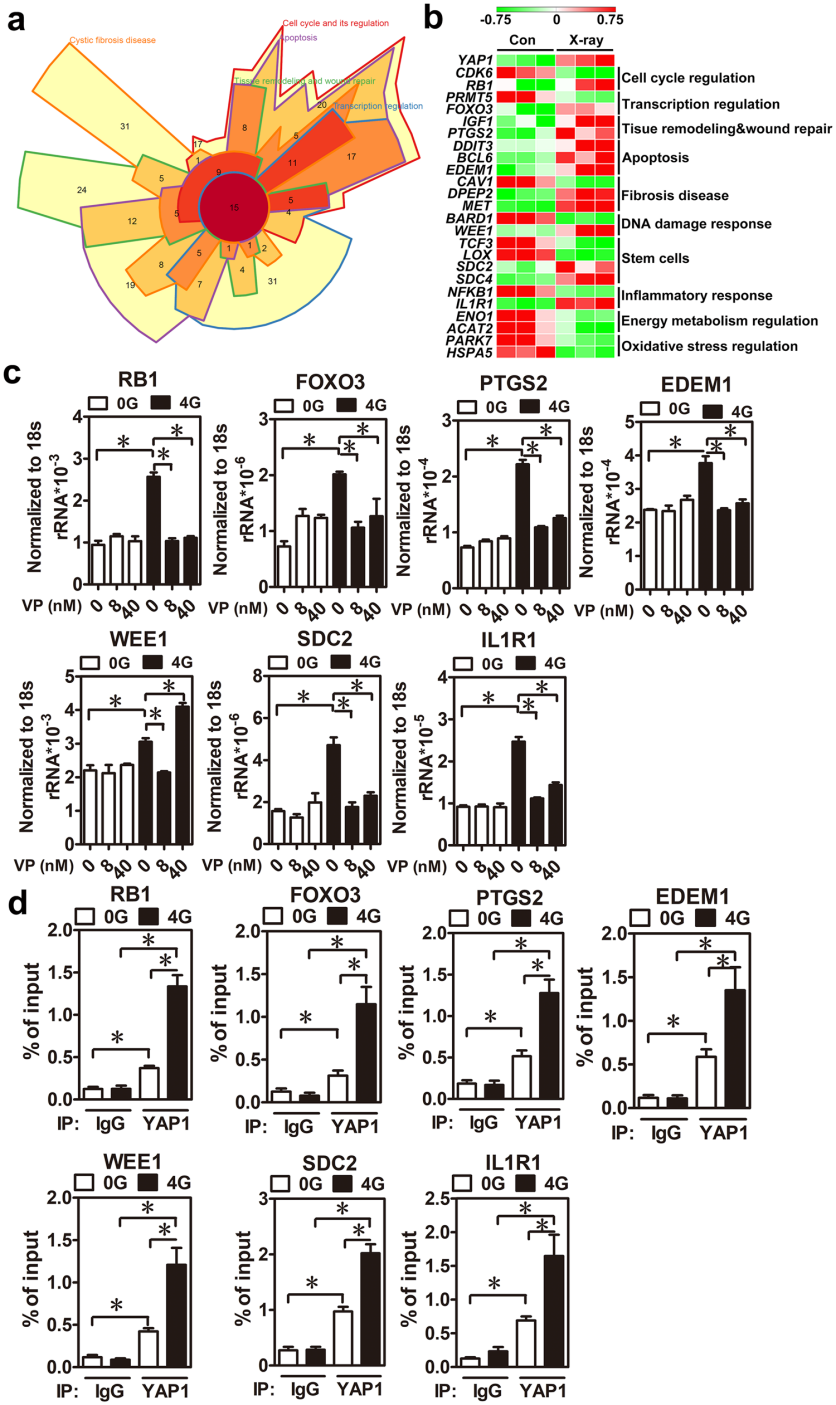
Numerous genes involved in different cellular processes of FICRD were directly regulated by YAP1 (**a**). Potential YAP1 downstream target genes involved in different cellular processes are presented as Venn diagrams using the Intervene tool. (**b**) Absolute changes in the gene expression profiles after radiation treatment were analyzed based on a GEO dataset (GSE23807) and presentated as heatmap. (**c**) HaCaT cells were treated with different doses of a VP compound for 24 h after radiation treatment. Several gene expression levels were measured using real-time PCR. The results were normalized to 18 s rRNA, which served as an internal control. The asterisk indicates *p* < 0.05 obtained using a paired test (*n* = 3). (**d**) HaCaT cells treated with radiation were incubated for 24 h, and ChIP-PCR was performed by using a YAP1 antibody. YAP1-binding profiles in different gene loci were detected using real–time PCR. An asterisk indicates *p* < 0.05 obtained using a paired test (*n* = 3)

To further investigate the role of YAP1 in MD tissue after radiation exposure, potential YAP1 downstream targets were re-analyzed from our previous study, and novel functions of YAP1 downstream targets not published in our previous study were identified [18]. The results showed that YAP1 downstream targets were involved in cell cycle regulation, tissue remodeling and wound repair, fibrosis, the DNA damage response, stem cells, and inflammation and oxidative stress responses (Fig. 4a). Furthermore, to validate the above findings, a public dataset (GSE23807) which performed a microarray analysis using a 3-dimensional skin model treated with 2.5 Gy irradiation for 24 h was reanalyzed by us. The heatmap results showed that the expression levels of YAP1 and its potential downstream target genes were statistically different in each category (Fig. 4b). To verify whether these potential YAP1 downstream target genes could be regulated by radiation through the YAP1 function, those gene expression profiles were analyzed in HaCaT cells received 4 Gy irradiation and combined with or without YAP1 inhibitor (verteporfin) treatment. The results showed that irradiation significantly increased RB1, FOXO3, PTGS2, EDEM1, WEE1, SDC2, and IL1R1 expression while the VP compound attenuated these gene expression levels under irradiation treatment (Fig. 4c). Furthermore, the YAP1 knockdown showed similar findings (Supplementary Fig. 4). Next, to investigate whether these potential downstream genes could be the direct targets of YAP1, YAP1-ChIP-seq results from the ReMap website were analyzed by us. Surprisingly, most of them contained YAP1-binding signals in their genomic loci (Supplementary Fig. 5). Next, irradiated-HaCaT cells were examined with ChIP-PCR using a YAP1 antibody. The results demonstrated that irradiation promoted YAP1 binding to its downstream targets (Fig. 4d). In summary, these findings suggest that YAP1 overexpression may regulate several crucial cellular processes through its downstream targets in response to radiation-induced skin damage.

### Exosomal YAP1 from irradiated keratinocytes attenuated fibroblast activation

**Fig. 5:**
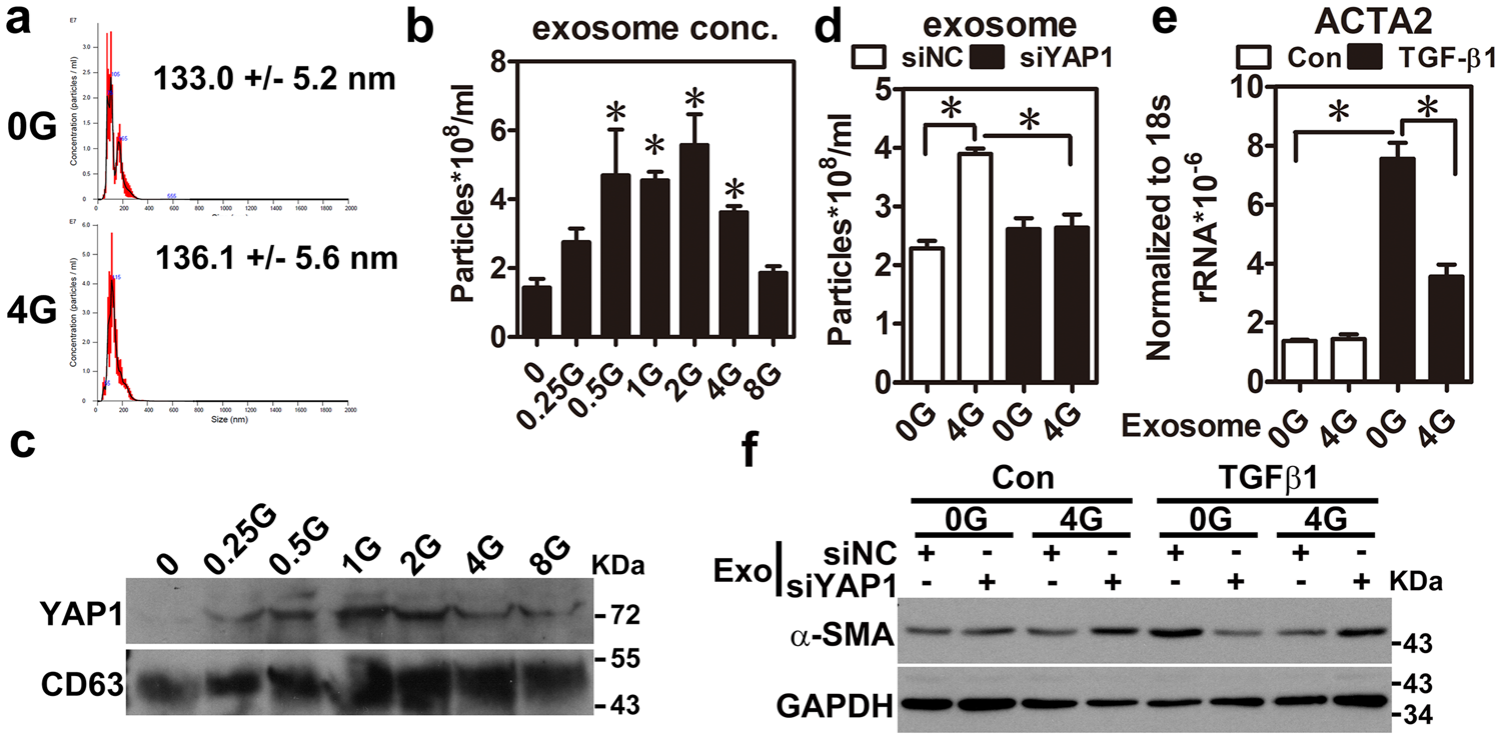
Radiation-induced exosome secretion inhibited myofibroblast activation via the function of YAP1. (**a**, **b**) Conditioned media from HaCaT cells treated with or without irradiation after incubation for 24 h were used for exosome isolation. Exosome sizes (**a**) and concentrations (**b**) were further analyzed using a nanoparticle tracking analysis (NTA). An asterisk indicates *p* < 0.05 using a one-way ANOVA test following a Dunnett’s analysis (*n* = 3). (**c**) YAP1 expression was measured in the exosomes isolated from the conditioned media of HaCaT cells treated with different doses of radiation using a western blot. CD63 was used as an exosome marker. (**d**) Exosome concentrations were detected in the HaCaT cells with (siYAP1) or without (siNC) YAP1 knockdown using an NTA analysis. An asterisk indicates *p* < 0.05 obtaining using a paired test (*n* = 3). (**e**) WS1 cells were pretreated with exosomes from HaCaT cells treated with or without irradiation for 24 h and then treated with TGF-β1 (5 ng/ml) for another 24 h. α-SMA expression was determined using a qRT-PCR. An asterisk indicates *p* < 0.05 obtained using a paired test (*n* = 3). (**f**) WS1 cells were pretreated with exosomes from HaCaT cells knocked down with or without YAP1 and treated with or without irradiation for 24 h, followed by treating with TGF-β1 (5 ng/ml) for another 24 h. α-SMA expression was determined using a western blot

Our current findings indicated that higher YAP1 expression in keratinocytes was associated with less fibroblast activation in the MD region, and the opposite finding was observed in the fibrotic region (Fig. 2c). Furthermore, both YAP1 mRNA and protein were identified in Vesiclepedia, a database of extracellular vehicles (EVs), suggesting it can be secreted into the extracellular microenvironment via EVs. Therefore, we hypothesized that radiation-induced YAP1 expression in keratinocytes could regulate fibroblast activation via remote control by EVs in the MD region. To test this idea, HaCaT cells were treated with different doses of radiation, and its culture media were used to isolate EVs using size exclusion chromatography. Interestingly, secretions of EVs were increased, but their size did not change after irradiation in HaCaT cells (Fig. 5a, b). The results also showed that YAP1 was detected in the EVs after irradiation (Fig. 5c). Furthermore, radiation-induced EV secretion was attenuated when YAP1 was knocked down by siRNA (Fig. 5d), suggesting that YAP1 may promote exosome secretion after irradiation. Next, WS1 cells were treated with EVs isolated from keratinocytes with or without radiation treatment. However, there were not apparent between-group differences in the fibroblast activation markers (Fig. 5e). Surprisingly, the EVs from the radiation-treated keratinocytes markedly reduced TGF-β1-induced ACTA2 (also called α smooth muscle actin, α-SMA), a marker of fibroblast activation, expression (Fig. 5e), revealing that it had the ability to suppress TGF-β1-induced fibroblast activation. More importantly, the above findings were attenuated when YAP1 in the EVs was primarily depleted by the pretreatment of siRNA against YAP1 in the HaCaT cells (Fig. 5f). Taken together, these results suggest that radiation-upregulated exosomal YAP1 inhibits TGF-β1-induced fibroblast activation in the MD region.

### Prednisolone is an effective drug with therapeutic potential for FICRD

**Fig. 6:**
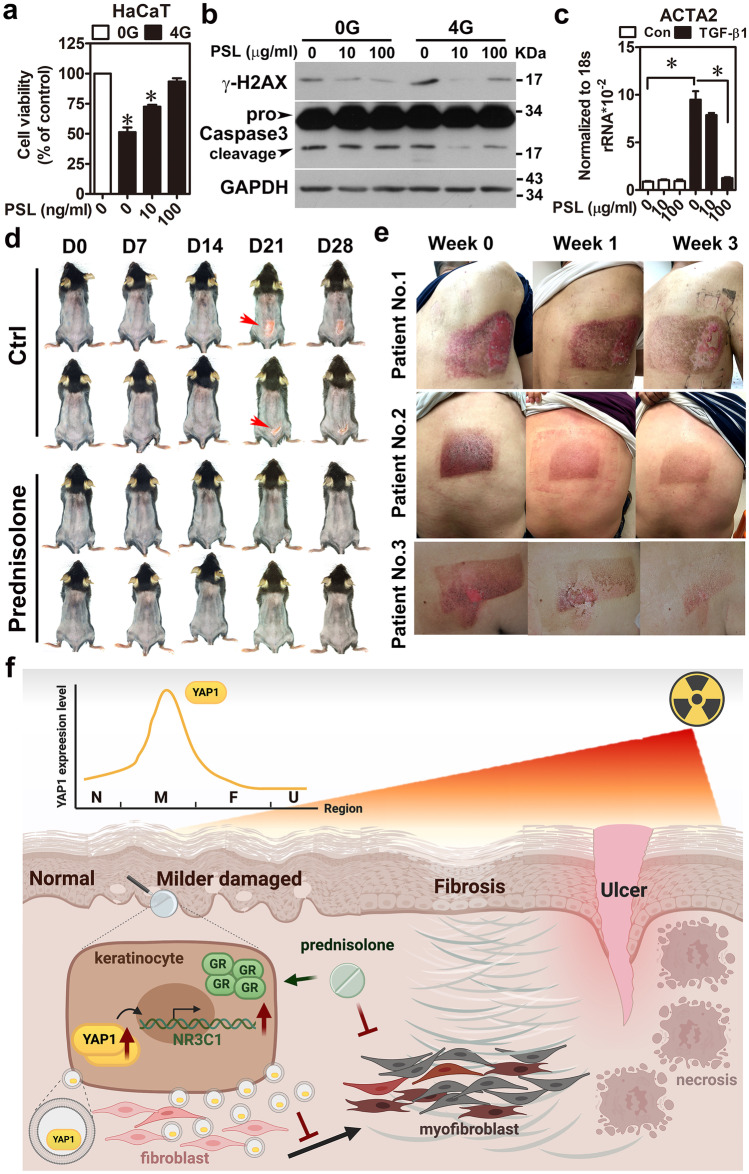
Prednisolone treatment prevented FICRD and attenuated the severity of FICRD (**a**, **b**). HaCaT cells were treated with different doses of prednisolone (PSL) for 24 h after irradiation. Cell viability (**a**) and expression levels of γ-H2AX and casepase-3 (**b**) were determined using an MTS assay and a western blot, respectively. An asterisk indicates *p* < 0.05 using a one-way ANOVA test following a Dunnett’s analysis (*n* = 3). (**c**) WS1 cells were pretreated with different doses of prednisolone (PSL) for 24 h and then treated with TGF-β1 (5 ng/ml) for another 24 h. An asterisk indicates *p* < 0.05 obtained using a paired test (*n* = 3). (**d**) Mice exposed to 30 Gy radiation received either a vehicle or prednisolone (0.5 mg/kg/day, 5 days per weeks) for 3 weeks after the 7th day following irradiation. Pictures of the skin of a mouse shown from the vehicle- and prednisolone-treated groups. The red arrow indicates the wounded region in the mouse skin. (**e**) These three patients had FICRD for more than 3 months, and conventional wound care failed to improve the skin disorder. They received oral prednisolone (initial dose as 5 mg, twice per day) for 3 weeks. Representative clinical pictures shown for different time courses after receiving prednisolone treatment. (**f**) An illustration briefly summarizing the role of YAP1 during the development of FICRD related to fluoroscopy-guided intervention. The figure was created using BioRender.com

Since there is no available approved therapy for FICRD, downstream target genes of YAP1 were analyzed to identify potential therapeutic targets. Among them, the NR3C1-encoded glucocorticoid receptor (GR) was selected because its available agonist and antagonist used in clinical practice and its expression level were elevated in the MD tissue compared to the normal counterpart (Supplementary Fig. 6a). In addition, we demonstrated that YAP1 directly increased NR3C1 expression in keratinocytes treated with radiation and fibroblasts co-treated with radiation and TGF-β1 (Supplementary Fig. 6b–d). Based on the above findings, prednisolone, a GR agonist, was used to investigate its effect on both keratinocytes and fibroblasts, as well as its therapeutic potential in patients with FICRD. First, HaCaT cells were co-treated using irradiation with or without prednisolone to determine cell survival, DNA damage, and apoptosis by performing a cell proliferation assay and measuring γ-H2AX and caspase-3 expression levels. The results showed that prednisolone not only promoted cell survival but also attenuated γ-H2AX and caspase-3 expression in the irradiated keratinocytes after 24 h (Fig. 6a, b). Similar results were observed at 72 h after the cells underwent irradiation (Supplementary Fig. 7a). Furthermore, prednisolone treatment also inhibited TGF-β1-induced activation of WS1 cells (Fig. 6c). To further investigate the therapeutic potential of prednisolone in vivo, a mouse model of FICRD was set up using irradiation on the back skin of a mouse. In the control group, skin ulcerations appeared on the 21st day after irradiation (Fig. 6d; Supplementary Fig. 7b). In the treatment group, prednisolone was intraperitoneally given five times a week for 1 month after the 7th day of irradiation. Surprisingly, prednisolone treatment prevented wound formation in the skin of the mouse exposed to irradiation (Fig. 6d; Supplementary Fig. 7b), indicating its therapeutic potential. Finally, the therapeutic efficacy of prednisolone was verified in patients with FICRD. They received oral 5 mg prednisolone twice per day for 3 weeks. The results showed that prednisolone led to a marked improvement in FICRD (Fig. 6e). Collectively, these results demonstrate that prednisolone is an appropriate therapeutic drug for treating FICRD.

## Discussion

FICRD could impose severe negative impacts on the health and quality of life of patients. It is very difficult to treat and might be overlooked because of its delayed onset and has a progressive deteriorating pathologic course. It appears initially as dermatitis, followed by fibrosis, with some eventually becoming unhealed ulcers. Although several potential therapies, including topical gels and photo-biomodulation, have been tested to determine their treatment efficacies [23, 24], there is no treatment proven to be effective as yet. Therefore, there is an urgent need for the development of an effective therapy for FICRD in clinical practice.

Herein, our study showed that YAP1 expression is specifically elevated in the mild damage zone of FICRD in patients (Fig. 2). Furthermore, radiation-induced YAP1 expression plays a protective role by promoting DNA damage repair in epidermal keratinocytes and in inhibiting dermal fibrosis in fibroblast cells after irradiation (Fig. 3). Previously, several studies revealed the crucial role of YAP1 in regulating chemotherapy-induced DNA damage repair [14, 15, 25]. However, an increase in RAD51, a gene involved in homologous recombination that is regulated by YAP1, is the only reported underlying mechanism explaining how YAP1 regulates DNA damage repair induced through cisplatin in breast cancer [25]. According to our findings, WEE1, a protein kinase involved in G_2_ cell cycle arrest by inactivating cyclin-dependent kinase 1-bound cyclin B in response to DNA damage, is positively regulated by YAP1 in irradiated skin cells (Fig. 4). In addition, several novel YAP1 downstream genes involved in different cellular processes were identified for the first time in the present study (Fig. 4). More importantly, NR3C1, one of the YAP1 downstream target genes, was shown to have therapeutic potential for FICRD since an agonist of NR3C1, prednisolone, attenuates its pathophysiologic changes in both animal model and human victims. (Fig. 6). Taken together, the present study is the first translational research to dissect the role of YAP1 in FICRD and to innovate a potential therapy for clinical application.

Corticosteroids are not expected as a treatment of choices for FICRD for several reasons. First, the role of inflammation in FICRD is not definite. There is a minimal inflammatory infiltrate in the histopathology of FICRD although there are clinical presentations of inflammation such as redness, local heat and pain [26]. It is unknown whether anti-inflammation is effective to treat FICRD or not. Second, it takes a long time to treat FICRD, since the course of FICRD is chronic and persistent. In order to suppress inflammation, a higher dose of systemic corticosteroids is required. A prolonged use of high dose of systemic corticosteroids poses negative impacts in patients’ health such as adrenal suppression, impaired immune function, and osteoporosis. If corticosteroids are used as an anti-inflammatory treatment for FICRD, a higher dose and a long treatment course should be given. It makes corticosteroids an unacceptable choice for treating FICRD. However, our experiences showed low dose of corticosteroids was very effective for FICRD both in preventing disease worsening and in relieving patients’ discomfort. Although there is no definite cut-point definition for “low” or “high” dose of steroids, it is worthily noted the dose of prednisolone used in this study could be tapered to as low as prednisolone 2.5 mg twice or trice per week within 2 months. According to knowledge derived from kidney transplant patients, long-term use of steroids (about 5–10 mg/day of prednisone) as maintenance therapy is required to abolish organ rejection. Such “physiologic” doses of steroids do not suppress adrenal or immune function, except affect metabolic function mildly [27].

It is important to treat not only dermal fibroblasts but also epidermal keratinocytes to attenuate ongoing fibrosis in FICRD. The interaction between the epidermis and dermis is crucial to maintain the integrity of skin function [28]. The functions of the epidermal keratinocytes include not only maintaining the skin barrier but also regulating the dermal fibroblasts underneath the skin [28, 29]. There is clinical evidence indicating that the epidermis can prevent or mitigate pathologic fibrosis in the dermis underneath. For example, treatment with topical emollients and silicone gel on the skin surface have been shown to effectively reduce the formation of keloids, hypertrophic scars, or radiotherapy-induced skin fibrosis [30]. Previous studies have shown that the interplay between epidermal keratinocytes and dermal fibroblasts is mediated by numerous soluble factors, including cytokines and growth factors, under different conditions [29, 31]. In the dermal fibrosis process, TGF-β1 represents a crucial soluble factor that promotes fibrosis through activation of fibroblasts [32]. Indeed, our results showed that TGF-β1 was increased in skin tissues with mild damage (Fig. 2a) and in irradiated-keratinocytes (Fig. 3c). Surprisingly, our findings revealed that irradiation stimulated exosome secretion in keratinocytes and repressed TGF-β1-induced fibroblast activation via the exosomal YAP1 function (Fig. 5). Recently, a study showed that irradiated-skin keratinocytes secrete miR-27a-containing exosomes and attenuate the migration ability of un-irradiated skin fibroblasts [33] Furthermore, exosomes from oral mucosal keratinocytes not only inhibit cell proliferation of skin fibroblasts but also promote wound healing [34]. Therefore, these findings indicate that irradiated epidermal keratinocytes may inhibit dermal fibroblast activation via exosomes and in turn prevent fibrosis. Taken together, our study demonstrated that keratinocytes with normal functions could attenuate and inhibit the progression of fibrosis via exosomal YAP1 function after exposure to radiation. In contrast, if keratinocytes are hampered with a higher dose of radiation that exceeds the threshold, they may fail to secrete exosomal YAP1 and fail to repress fibrosis while promoting fibrosis via the TGF-β1 pathway. More importantly, targeting NR3C1, a YAP1 downstream target, may be a potential therapy for the treatment of FICRD in the future (Fig. 6f).

## Supplementary Information

Below is the link to the electronic supplementary material.

Supplementary file1 (DOCX 2303 KB)

Supplementary file2 (DOCX 20 KB)

## Data Availability

The generated dataset and material used in the current study are available from the corresponding author.

## References

[1] Ryan JL (2012). Ionizing radiation: the good, the bad, and the ugly. J Invest Dermatol.

[2] Koenig TR, Wolff D, Mettler FA, Wagner LK (2001). Skin injuries from fluoroscopically guided procedures: part 1, characteristics of radiation injury. AJR Am J Roentgenol.

[3] Benjamin EJ, Muntner P, Alonso A, Bittencourt MS, Callaway CW, Carson AP, Chamberlain AM, Chang AR, Cheng S, Das SR (2019) Heart disease and stroke Statistics-2019 update a report from the American Heart Association. Circulation 10.1161/CIR.0000000000000659 30700139

[4] Balter S, Miller DL (2014). Patient skin reactions from interventional fluoroscopy procedures. AJR Am J Roentgenol.

[5] Hegedus F, Mathew LM, Schwartz RA (2017). Radiation dermatitis: an overview. Int J Dermatol.

[6] Wei KC, Yang KC, Chen LW, Liu WC, Chen WC, Chiou WY, Lai PC (2016). Management of fluoroscopy-induced radiation ulcer: one-stage radical excision and immediate reconstruction. Sci Rep.

[7] Koenig TR, Mettler FA, Wagner LK (2001). Skin injuries from fluoroscopically guided procedures: part 2, review of 73 cases and recommendations for minimizing dose delivered to patient. AJR Am J Roentgenol.

[8] Bruskov VI, Karp OE, Garmash SA, Shtarkman IN, Chernikov AV, Gudkov SV (2012). Prolongation of oxidative stress by long-lived reactive protein species induced by X-ray radiation and their genotoxic action. Free Radic Res.

[9] Derheimer FA, Kastan MB (2010). Multiple roles of ATM in monitoring and maintaining DNA integrity. FEBS Lett.

[10] Rizzo AM, Berselli P, Zava S, Montorfano G, Negroni M, Corsetto P, Berra B (2010). Endogenous antioxidants and radical scavengers. Adv Exp Med Biol.

[11] Flynn RL, Zou L (2011). ATR: a master conductor of cellular responses to DNA replication stress. Trends Biochem Sci.

[12] Iyama T, Wilson DM (2013). DNA repair mechanisms in dividing and non-dividing cells. DNA Repair (Amst).

[13] Rowe LA, Degtyareva N, Doetsch PW (2012). Yap1: a DNA damage responder in *Saccharomyces cerevisiae*. Mech Ageing Dev.

[14] Ciamporcero E, Shen H, Ramakrishnan S, Yu KuS, Chintala S, Shen L, Adelaiye R, Miles KM, Ullio C, Pizzimenti S (2016). YAP activation protects urothelial cell carcinoma from treatment-induced DNA damage. Oncogene.

[15] Andrade D, Mehta M, Griffith J, Panneerselvam J, Srivastava A, Kim TD, Janknecht R, Herman T, Ramesh R, Munshi A (2017). YAP1 inhibition radiosensitizes triple negative breast cancer cells by targeting the DNA damage response and cell survival pathways. Oncotarget.

[16] Blanpain C, Fuchs E (2006). Epidermal stem cells of the skin. Annu Rev Cell Dev Biol.

[17] Schlegelmilch K, Mohseni M, Kirak O, Pruszak J, Rodriguez JR, Zhou D, Kreger BT, Vasioukhin V, Avruch J, Brummelkamp TR (2011). Yap1 acts downstream of alpha-catenin to control epidermal proliferation. Cell.

[18] Lin SC, Lee HC, Hou PC, Fu JL, Wu MH, Tsai SJ (2017). Targeting hypoxia-mediated YAP1 nuclear translocation ameliorates pathogenesis of endometriosis without compromising maternal fertility. J Pathol.

[19] Gregorieff A, Liu Y, Inanlou MR, Khomchuk Y, Wrana JL (2015). Yap-dependent reprogramming of Lgr5(+) stem cells drives intestinal regeneration and cancer. Nature.

[20] Elbediwy A, Vincent-Mistiaen ZI, Spencer-Dene B, Stone RK, Boeing S, Wculek SK, Cordero J, Tan EH, Ridgway R, Brunton VG (2016). Integrin signalling regulates YAP and TAZ to control skin homeostasis. Development.

[21] Andl T, Zhou L, Yang K, Kadekaro AL, Zhang Y (2017). YAP and WWTR1: New targets for skin cancer treatment. Cancer Lett.

[22] Qin Z, Xia W, Fisher GJ, Voorhees JJ, Quan T (2018). YAP/TAZ regulates TGF-beta/Smad3 signaling by induction of Smad7 via AP-1 in human skin dermal fibroblasts. Cell Commun Signal.

[23] Iacovelli NA, Naimo S, Bonfantini F, Cavallo A, Bossi P, Fallai C, Pignoli E, Alfieri S, Bergamini C, Favales F (2017). Preemptive treatment with Xonrid(R), a medical device to reduce radiation induced dermatitis in head and neck cancer patients receiving curative treatment: a pilot study. Support Care Cancer.

[24] Park JH, Byun HJ, Kim HJ, Oh SJ, Choi C, Noh JM, Oh D, Lee JH, Lee DY (2020) Effect of photobiomodulation therapy on radiodermatitis in a mouse model: an experimental animal study. Lasers Med Sci. 10.1007/s10103-020-03123-x 10.1007/s10103-020-03123-x 32876761

[25] Elaimy AL, Amante JJ, Zhu LJ, Wang M, Walmsley CS, FitzGerald TJ, Goel HL, Mercurio AM (2019). The VEGF receptor neuropilin 2 promotes homologous recombination by stimulating YAP/TAZ-mediated Rad51 expression. Proc Natl Acad Sci USA.

[26] Liao JB, Chen W, Lee HS, Wu SR, Wei KC (2020). Histopathology of fluoroscopy-induced radiation ulcer: a case series study in comparison with morphea. J Dtsch Dermatol Ges.

[27] Steiner RW, Awdishu L (2011). Steroids in kidney transplant patients. Semin Immunopathol.

[28] Rippa AL, Kalabusheva EP, Vorotelyak EA (2019). Regeneration of dermis: scarring and cells involved. Cells.

[29] Russo B, Brembilla NC, Chizzolini C (2020) Interplay between keratinocytes and fibroblasts: a systematic review providing a new angle for understanding skin fibrotic disorders. Front Immunol 11 10.3389/fimmu.2020.00648 PMC7232541 32477322

[30] Tziotzios C, Profyris C, Sterling J (2012) Cutaneous scarring: pathophysiology, molecular mechanisms, and scar reduction therapeutics Part II. Strategies to reduce scar formation after dermatologic procedures. Journal of the American Academy of Dermatology 66: 13–24; quiz 25–16. 10.1016/j.jaad.2011.08.035 10.1016/j.jaad.2011.08.035 22177632

[31] Sorrell JM, Baber M, Caplan A (2004). Site-matched papillary and reticular human dermal fibroblasts differ in their release of specific growth factors/cytokines and in their interaction with keratinocytes. J Cell Physiol.

[32] Martin M, Lefaix J, Delanian S (2000). TGF-beta1 and radiation fibrosis: a master switch and a specific therapeutic target?. Int J Radiat Oncol Biol Phys.

[33] Tan W, Zhang Y, Li M, Zhu X, Yang X, Wang J, Zhang S, Zhu W, Cao J, Yang H (2019). miR-27a-containing exosomes secreted by irradiated skin keratinocytes delayed the migration of unirradiated skin fibroblasts. Int J Biol Sci.

[34] Sjoqvist S, Ishikawa T, Shimura D, Kasai Y, Imafuku A, Bou-Ghannam S, Iwata T, Kanai N (2019). Exosomes derived from clinical-grade oral mucosal epithelial cell sheets promote wound healing. J Extracell Vesicles.

