# Supplementary material for: An innovative targeted therapy for fluoroscopy-induced chronic radiation dermatitis
Source: J Mol Med (Berl). 2021 Oct 23;100(1):135–46. doi: 10.1007/s00109-021-02146-3 (PMC8724166; doi:10.1007/s00109-021-02146-3)
Supplement: Supplementary file 1 — Supplementary file1 (DOCX 2303 KB) [file 109_2021_2146_MOESM1_ESM.docx]

**
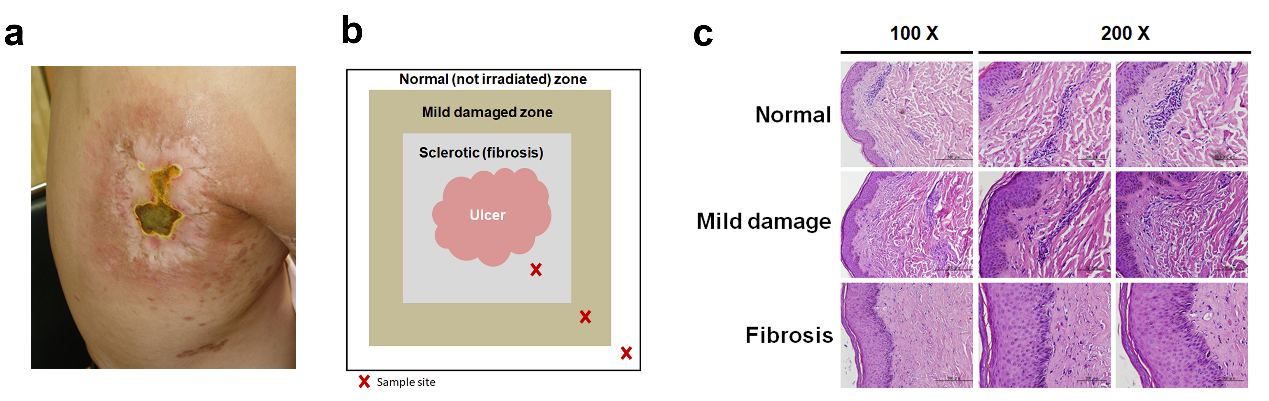
Supplementary data**

**Supplementary Fig. 1: Pathological profiles of collecting skin tissues from a patient with FICRD. (a)** Representative picture FICRD from a 56-year-old male patient. **(b)** Cartoon indicates the sample regions including normal, mild damaged and fibrotic tissues were collected for further analyses. **(c)** Hematoxylin and eosin (H&E) staining shows the compositions from different sample regions.


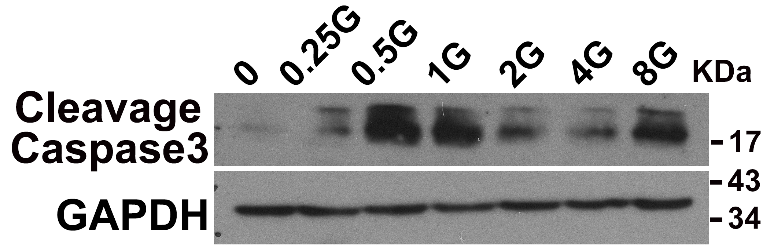


**Supplementary Fig. 2: Radiation treatment increased level of cleavage casepase-3 after incubation for longer time point.** Casepase-3 expression levels were analyzed in HaCaT cells treated with different doses of irradiation after incubation for 72 hours.


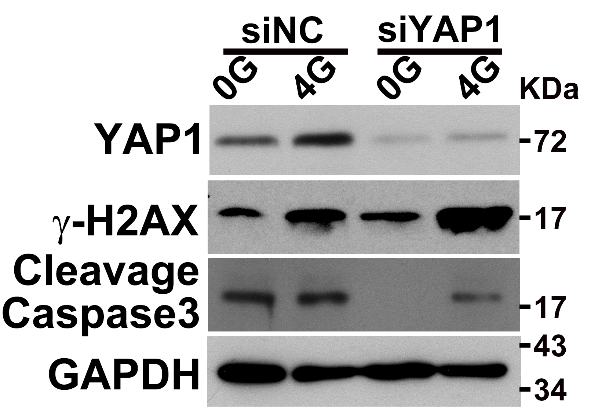


**Supplementary Fig. 3: YAP1 knockdown further increased expression levels of DNA damage and apoptosis markers after radiation treatment in HaCaT cells.** HaCaT cells were knocked down YAP1 expression by siRNA for 24 hours and then incubated for another 24 hours after radiation treatment. YAP1, γ-H2AX and cleavage casepase-3 expression levels were analyzed by Western blot.


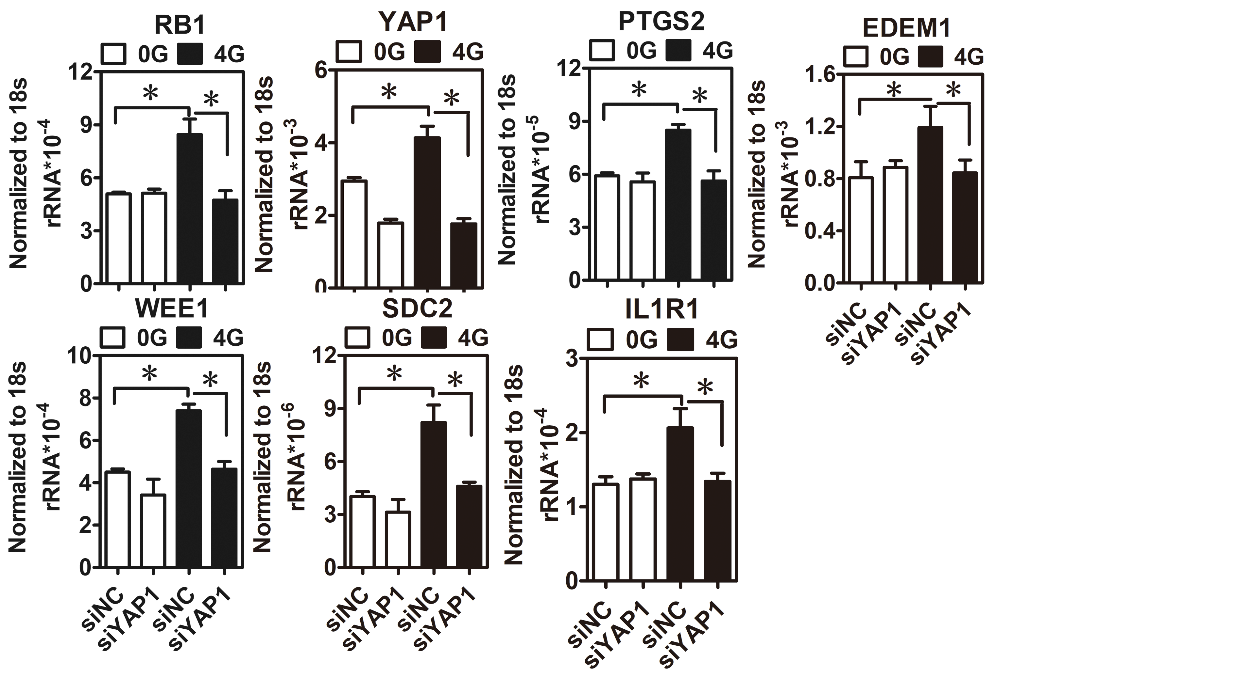
**Supplementary Fig. 4: Numerous genes involved in different cellular processes of radiation dermatitis were directly regulated by YAP1**. HaCaT cells were treated with control or siRNA against YAP1 for 24 hours and then incubated for another 24 hours after radiation treatment (0G and 4G). Several gene expression levels were measured by real-time PCR. Results were normalized to 18s rRNA, an internal control. Asterisk indicates p < 0.05 by using paired test (n=3).
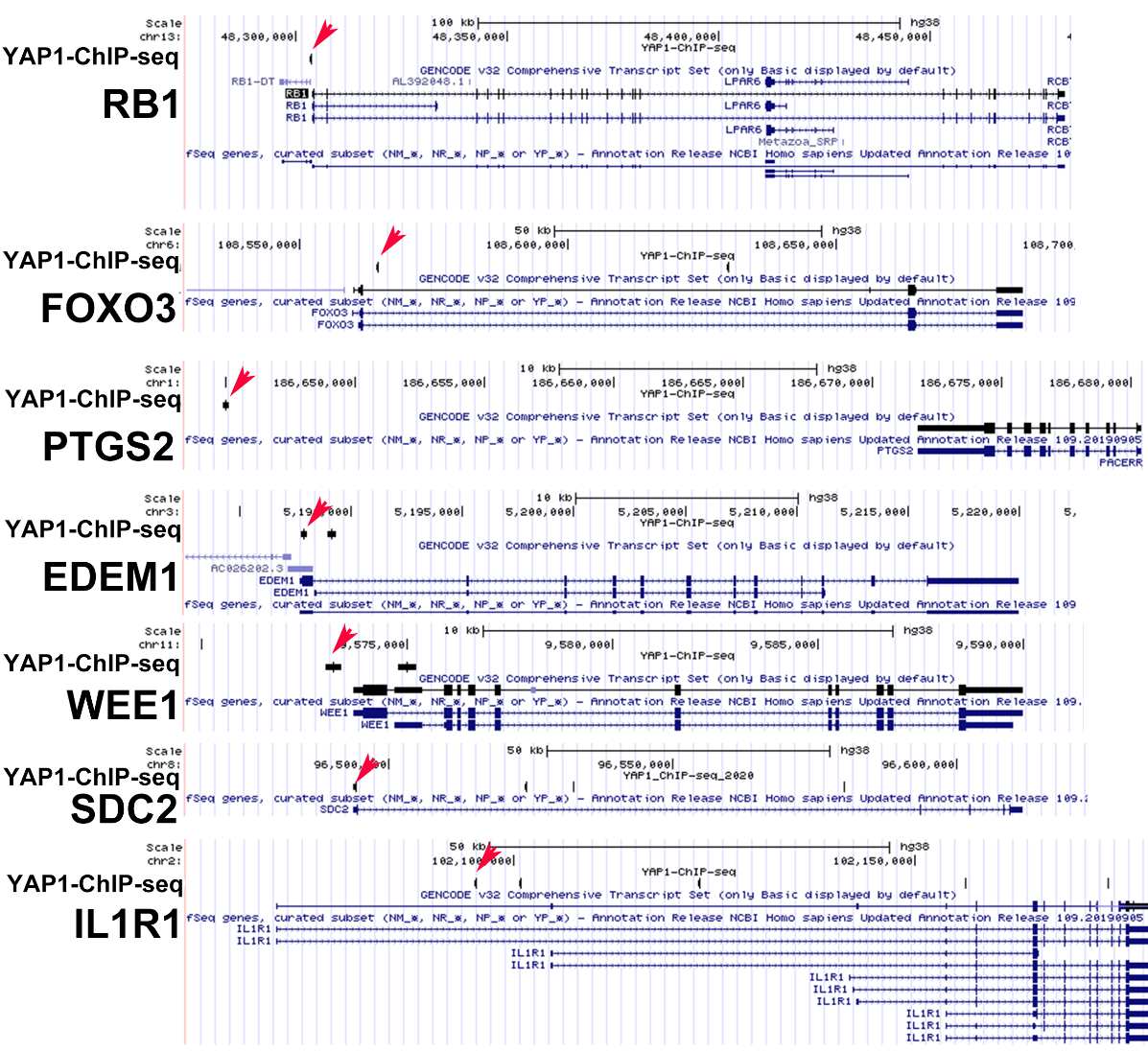


**Supplementary Fig. 5: Potential YAP1 downstream target genes had YAP1 binding signals in their gene loci**. YAP1-ChIP-seq result was analyzed from ReMap2020 (<http://remap.univ-amu.fr/>). Genes with YAP1 binding peaks were presented and red arrows indicated YAP1 binding regions in those gene loci.


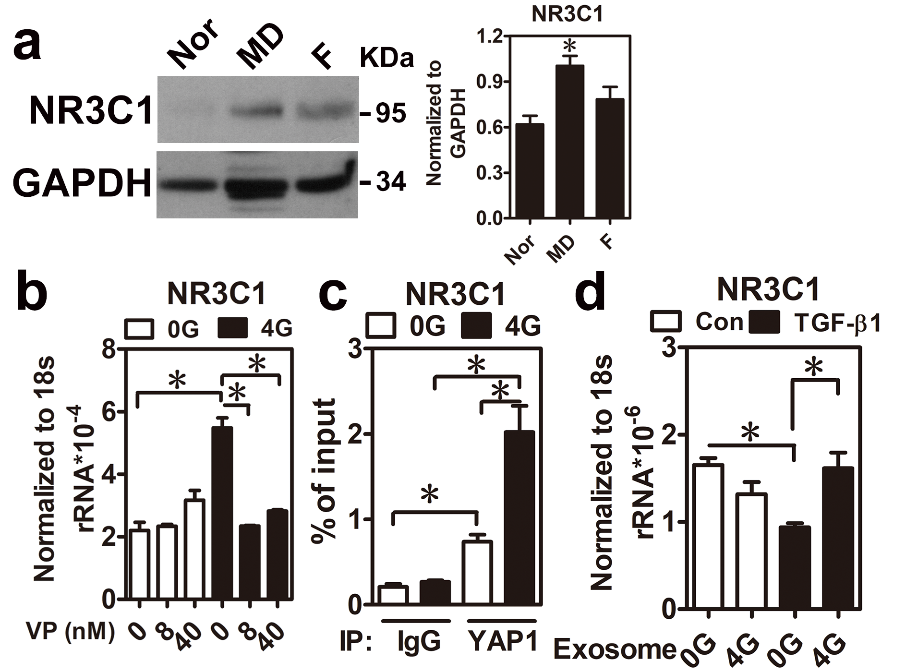


**Supplementary Fig. 6: Overexpression of NR3C1 in the skin tissue from a patient with FICRD was positively regulated by YAP1.** **(a)** NR3C1 expression in the skin tissue samples from normal, mild damage and fibrosis were measured by Western blot (left panel). Quantification result was presented in the right panel (n=3). Asterisk indicates p < 0.05 by using one-way ANOVA test following Dunnett’s analysis. **(b)** HaCaT cells were treated with different doses of VP compound for 24 hours after radiation treatment. NR3C1 expression level was measured by real-time PCR. Resutls were normalized to 18s rRNA, an internal control. Asterisk indicates p < 0.05 by using paired test (n=3). **(c)** HaCaT cells treated with radiation was incubated for 24 hours and performed ChIP-PCR by using YAP1 antibody. YAP1-binding profile in NR3C1 gene locus was detected by real-time PCR. Asterisk indicates p < 0.05 by using paired test (n=3). **(d)** WS1 cells were pretreated with exosomes from HaCaT cells treated with or without radiation for 24 hours and then treated with TGFb1 (5ng/ml) for another 24 hours. NR3C1 expression was determined by realtime PCR. Results were normalized to 18s rRNA. Asterisk indicates p < 0.05 by using paired test (n=3).


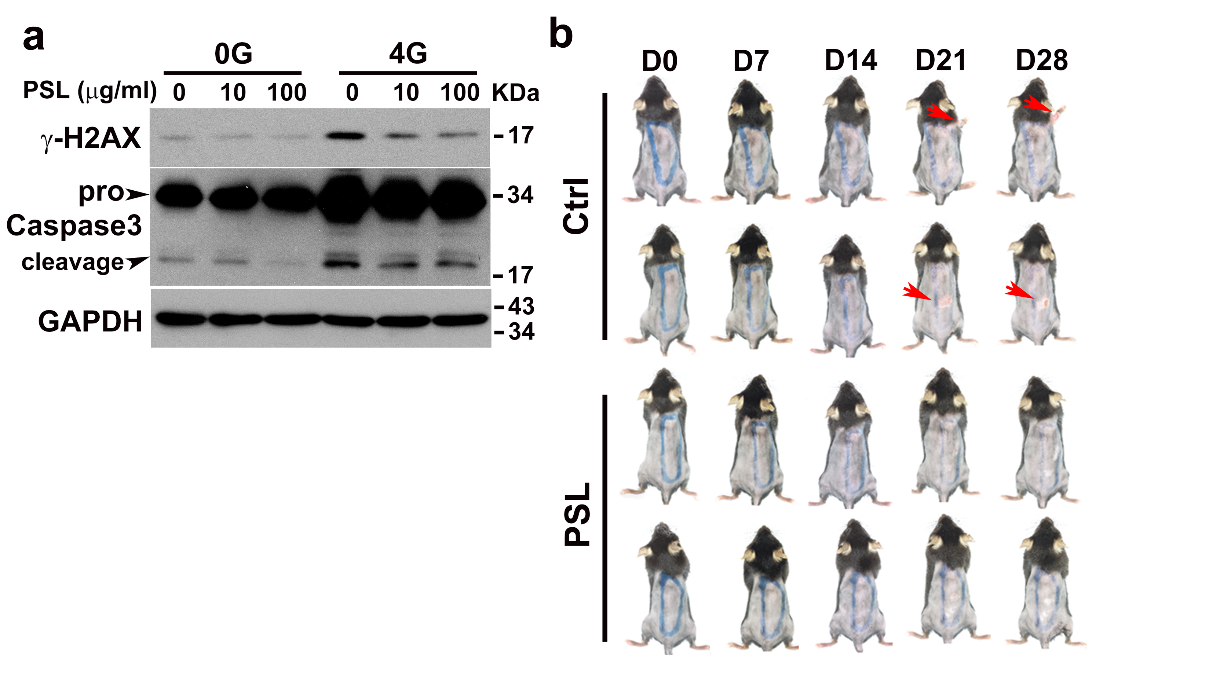
**Supplementary Fig. 7: Prednisolone showed the therapeutic potential in vitro and in vivo. (a)** HaCaT cells were treated with different doses of prednisolone for 72 hours after radiation treatment. Expression levels of γ-H2AX and casepase-3 were determined by Western blot. **(b)** Mice exposed to 30 Gy radiation were received vehicle or prednisolone (0.5 mg/day, 5 days per weeks) for three weeks since the 7th day after irradiation. Pictures of mouse skin were shown from the vehicle and prednisolone treatment groups. Red arrow indicated wounded region in the mouse skin.

**Supplementary tables:**
