# Supplementary material for: An innovative targeted therapy for fluoroscopy-induced chronic radiation dermatitis
Source: J Mol Med (Berl). 2021 Oct 23;100(1):135–46. doi: 10.1007/s00109-021-02146-3 (PMC8724166; doi:10.1007/s00109-021-02146-3)
Supplement: Supplementary file 2 — Supplementary file2 (DOCX 20 KB) [file 109_2021_2146_MOESM2_ESM.docx]

**Supplementary materials and methods**

**Patients and clinical specimens**

The patients diagnosed with FICRD at Kaohsiung Veterans General Hospital between 2012 and 2019 were included for the purpose of analyzing their medical data, including the clinical responses toward treatment during the clinical follow-up. Skin specimens were obtained during radical excision and reconstruction, which was indicated when patients’ radiation ulcer was refractory to treatment, including conventional wound care and hyperbaric oxygen therapy for more than 3 months and effects on the patients’ quality of life. For treating patients with FICRD, the initial dosage of oral prednisolone was 10 mg daily. The dose would be adjusted based on severity of the clinical symptoms since the 3rd week and could be tapered to as low as prednisolone 2.5 mg twice or trice per week within 2 months.

**Cell culture and treatment**

Human keratinocyte HaCaT cells (Cell Lines Service, CLS) were grown in Dulbecco’s modified Eagle’s medium (DMEM) containing with 10% of heat-inactivated fetal bovine serum (FBS, Gibco) and 1% antibiotic-antimycotic (Gibco). WS1, a normal human skin fibroblast cell line (Bioresource Collection and Research Center of Hsinchu in Taiwan), cells were cultured in the Minimum Essential Media (MEM) with 10% FBS at 37°C and 5% CO_2_. HaCaT cells or W1 cells on coverslips were irradiated with 0, 1, 2, 4, 8, 16 Gy of gamma rate at room temperature after plating for 24 hours. Then, HaCaT cells were replenished with fresh medium. For YAP1 inhibitor treatment, HaCaT cells were changed to serum free medium before irradiation. HaCaT cells were treated with different concentrations of YAP1 inhibitors after irradiation for different doses (0, 1, 2 and 4-Gy). Then, HaCaT cells were harvested for RNA and protein isolations after treatment for 24 hours.

**RNA isolation and detection**

Cells and tissues were separately collected for RNA isolation by using TRIzol reagent (Invitrogen, USA) according to protocol provided by manufacture. A NanoDrop 2000 (Thermo Fisher Scientific, Waltham, MA, USA) was used to evaluate the quantity of RNA, and an Agilent 2100 bioanalyzer (Agilent Technologies, Santa Clara, CA) was used to assess RNA quality. Next, total RNA was performed reverse transcription by M-MLV reverse transcriptase (Promega Corporation, WI, USA) to generate complementary DNA (cDNA). Specific gene expression profiles were measured by StepOnePlus Real-Time PCR system. Primer sequences for individual genes were listed in the **Supplementary Table 2**.

**Western blot**

Protein concertation was determined by Pierce™ BCA Protein Assay Kit (Thermo Fisher Scientific, Waltham, MA, USA). Protein samples (30 μg) were separated by SDS-PAGE and transferred to the PVDF membrane. Membrane was further blocked by 5% non-fat milk at room temperature for one hour and incubated overnight at 4°C by using different first antibodies. Second antibodies were prepared in the 5% non-fat milk and added to the PVDF membrane at room temperature for one hour. Next, membrane was washed by 0.05% PBST buffer for another one hour. Finally, membrane was treated by Pierce™ ECL Western blotting substrate (Thermo Fisher Scientific, Waltham, MA, USA) and exposed to the X-ray film. Antibody information was listed in the **Supplementary Table 3**.

**Immunohistochemistry (IHC) staining**

Skin specimens of radiation-induced dermatitis including normal, milder damaged and fibrotic skin tissues were analyzed YAP1 protein expression and distribution by IHC staining. Paraffin-embedded tissue samples were performed procedures of deparaffinization, rehydration, antigen retrieval and inactivation of endogenous peroxidase according to the standard protocol. Signal detection was used DAB system. YAP1 antibody information was listed in the **Supplementary Table 3**.

**Cell viability assays**

HaCaT cells (5x10^3^) were seeded in 96-well plates containing DMEM medium supplemented with 10% FBS and incubated at 37°C, 5% CO_2_ overnight. Next, HaCaT cells were replaced with serum free medium before irradiation. Then, HaCaT cells were treated with 0, 0.5, 1, 2, 4, 8 and 16-Gy of irradiation and incubated for 24, 48 and 120 hours. Cell viability was detected by using a CellTiter-Glo Luminescent cell viability assay (cat. G7570, Promega) according to the manufacturer’s instruction.

***Small interference RNA (siRNA)***

Control and siRNA against YAP1 were purchased from Invitrogen Life Technologies (Carlsbad, CA, USA). HaCaT cells were transfected with control or YAP1 siRNA (20 nM) by using lipofectamine 2000 (Invitrogen) according to manuscript’s protocol for 16 hours. After incubation, culture medium was changed to serum free medium and then HaCaT cells were irradiated with 0, 1, 2 and 4-Gy irradiation at room temperature. HaCaT cells were harvested by TRIzol regagent after treatment for 24 hours. Sequence of siRNA against YAP1 was listed in the antibody information was listed in the **Supplementary Table S2**.

**Chromatin immunoprecipation assay (ChIP)**

HaCaT cells (2x10^6^) were seeded and changed to serum free medium before irradiation. Next, HaCaT cells were irradiated with 0, 1, 2 and 4-Gy dose and incubated for 24 hours. Cells were fixed by 1% paraformaldehyde for 10 min and cross-linking reaction was stopped by adding 0.125 M glycine solution for 10 min at RT before sample collection. Then, samples were performed ChIP analysis by using Pierce™ Magnetic ChIP Kit (Thermo Fisher Scientific, Waltham, MA, USA) and according to protocol provided by manufacture. Next, ChIP samples were further analyzed by quantitative real-time PCR by specific primers. Primer sequences and antibody were listed in the **Supplementary Table 2&3**, respectively.

**NGS (Next Generation Sequencing) and bioinformatics analyses**

Total RNA from skin specimens of radiation-induced dermatitis including normal, milder damaged and fibrotic skin tissues were used to perform NGS analysis. RNA integrity was estimated by microfluidic capillary electrophoresis by running 5 μl of each sample on an Agilent 2100 bioanalyzer system (Agilent Technologies, Santa Clara, CA, USA) using Agilent RNA 6000 Pico kit. The concentration of RNA was quantified with Qubit ® RNA HS assay kit on a Qubit 2.0 Fluorometer (Thermo Fisher Scientific, Waltham, MA, USA. RNA was performed for library preparation using TruSeq® RNA Access Library Preparation kit (Illumina, San Diego, CA, USA) in accordance with the manufacturer's instructions. The cluster of libraries were sequenced on the Illumina HiSeq 2500 platform (Illumina®) with a rapid paired-end sequencing (2 x 101 bp). The libraries generation were determined by adjustment of cDNA amount used for hybridization. Gene lists from different regions of radiation-induced dermatitis were further analyzed by gene ontology (GO) analysis provided from the DAVID, a bioinformatics tool.

**Exosome isolation and analysis**

HaCaT cells (2x10^6^) were seeded and changed to a serum-free medium before irradiation. They were irradiated with a series of doses of irradiation and incubated for 24 hours. Next, conditioned media was collected and centrifuged 2000 хg for 5 min to remove cellular debris. Exosomes were isolated from the conditioned medium using the ExoQuick™ exosome precipitation solution (System Biosciences, Palo Alto, CA). Next, the exosome sizes and concentrations were calculated using NanoSight LM10-HS (Malvern Panalytical, Malvern, UK) in the Center for Micro/Nano Science and Technology at National Cheng Kung University.

**Co-culture system**

Transwell migration assay utilizing 24-well hanging inserts fitted with an 8 μm pore size membrane (Millicell cell culture inserts cat. MCEP24H48) was used to study the interaction between HaCaT cells and WS1, a fibroblast cell lin. HaCaT cells (5x10^4^) were seeded onto the apical surface of each hanging-insert and irradiated with 0 and 4-Gy under serum-free condition. Hanging-inserts containing irradiated-HaCaT cells were moved to a 24 well plate containing WS1 cells (2.5x10^4^ cells/well). Next, TGF-β1 (5 ng/ml) was added to the culture plate under serum-free condition and incubated for 24 hours. WS1 cells were collected for RNA isolation by using TRIzol reagent.

**Animal model of irradiation**

All mice were housed and maintained in the animal facility of National Taiwan University (Taipei, Taiwan). Female C57BL/6 mice (12-wk-old) were purchased from the Taiwan National Laboratory Animal Center. Mice were anesthetized by intramuscular injection of a 4:1 mixture (v/v) of zolazepam (Zoletilt; Virbac Animal Health, Inc., Fort Worth, TX) and xylazine (Rompune; Bayer HealthCare LLC, Whippany, NJ) for experiments. Hairs on the back of 12-week-old female mice were carefully shaved without injuring the skin before irradiation. Mice were anesthetized prior to irradiation by a cesium-137 source (IBL-637 gamma irradiator, 662 keV photons, CIS Bio International, Gif sur Yvette, France). Both control and treatment groups were exposed to 30 Gy irradiation from the dorsal side of back. In the treatment group, intraperitoneal administration of prednisolone 0.5 mg/kg/day since the 7^th^ after irradiation was given daily for 5 days per week for the following 3 weeks. After irradiation, we kept daily observation of clinical expression of any inflammation and skin defects. And then, skin specimens were sampled at 28 days post-irradiation for analysis.
